# A Review Of Oculoplastic Photography: A Guide For Clinician Photographers

**DOI:** 10.7759/cureus.733

**Published:** 2016-08-11

**Authors:** Chin T Ong, Jun Fai Yap, Yong Zheng Wai, Qi Xiong Ng

**Affiliations:** 1 Ophthalmology, Beacon International Specialist Hospital; 2 Ophthalmology, University of Malaya

**Keywords:** clinical photography, standardization, oculoplastic surgery

## Abstract

Clinical photography in the field of oculoplastic surgery has many applications. It is possible for clinicians to obtain standardized clinical photographs without a studio. A clinician photographer has the advantage of knowing exactly what to photograph as well as having immediate access to the images. In order to maintain standardization in the photographs, the photographic settings should remain constant. This article covers essential photographic equipment, camera settings, patient pose, and digital asset management.

## Introduction and background

Clinical photographs are useful for clinical records, research, audit, education, and in supporting medico-legal cases [1-3]. Standardized clinical photographs are often taken by medical photographers using professional grade photographic equipment in dedicated studios. However, it is possible for clinicians to obtain high quality clinical photographs using simpler photographic equipment outside a studio [4]. There are many benefits if a clinician takes patient photographs. The clinician photographer knows without instructions exactly which area of the body to photograph. The photographic session can take place during the patient consultation with no further delays for the patient [1]. The images are instantly available to the clinician and can be shown to the patient during the consultation process.

### Standardization

The most important concept in oculoplastic photography is standardization [1, 4-5]. The only parameter that changes over time is the patient while the photographic set up remains constant. In practice this means that the pose of the patient, focal length of the lens, image magnification, aperture setting, shutter speed, ISO, file size, file types, lighting condition, background, and white balance must remain constant from shot to shot [6-8]. A standardized photograph enables an accurate measurement of treatment over time.

### Legal and ethical considerations in external clinical photography

It is important to check the local data protection regulations before processing personal and sensitive data that includes photographs [2]. Ideally, written informed consent must be obtained prior to photography. The data needs to be securely stored, kept up-to-date, and backed up regularly in line with the data protection laws.

## Review

### Photographic setup for standardized representational photography in oculoplastic surgery

Any camera can be used in the clinical setting to create a snapshot of a patient. However, in order to achieve image standardization, a very specific set of photographic equipment is required.

There are three main aspects to consider when shopping for a camera, namely the image sensor size, lens focal length, and lighting options [3].

The choice of camera depends on the lens-sensor combination. Correct lens focal length and sensor size combination are important in maintaining standardization of perspective [9]. The focal length of the lens has to be at least twice the diagonal dimension of the sensor to achieve normal perspective. Any other lens-sensor combination will introduce some degree of distortion to the final image.

### Image sensors

**Figure 1:**
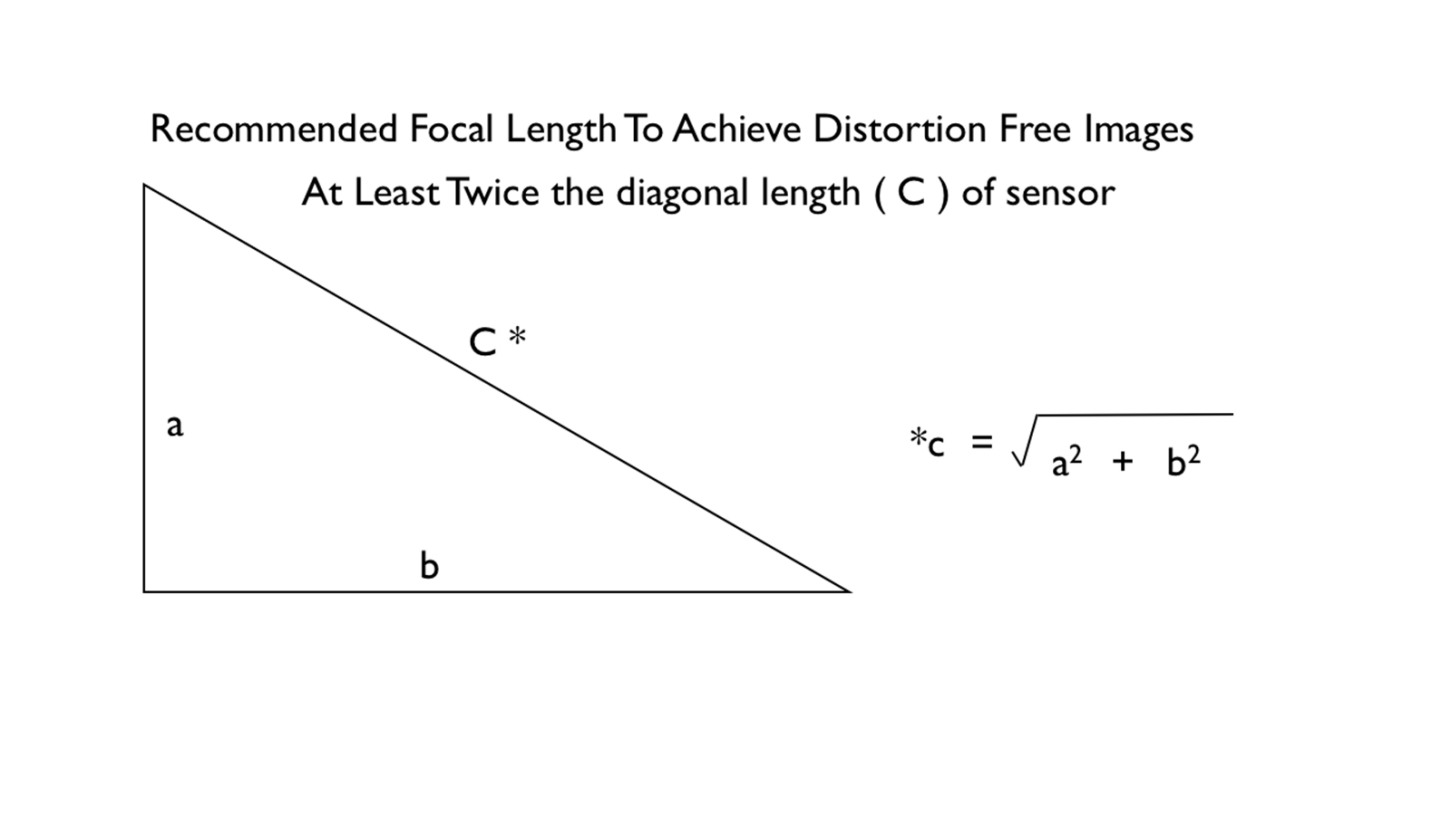
Recommended Focal Length

The ideal camera is a digital camera equipped with a large image sensor. The most common type of image sensor is the Advanced Photographic System type C (APS-C) sensor. The dimension of an APS-C sensor is typically 22.3 mm x 14.9 mm (Canon). Top-of-the-range digital cameras are often equipped with a full-frame sensor measuring 24 mm x 35 mm. This is significantly larger than the APS-C sensor. A large sensor camera has the advantage of producing better quality images with more details and less digital noise. However, full-frame cameras are usually heavier and more expensive. The choice of lens focal length will depend on the sensor size in order to minimise distortion of perspective. As a rule the ideal focal length of a lens should be at least twice the diagonal dimension of the sensor, as illustrated in (Figure 1) [9]. 

For example, the diagonal dimension of a Canon APS-C sensor is 26.81 mm. This means the ideal lens focal length is 54 mm. In practice, the most commonly used focal length is 60 mm. When a full-frame sensor (24 mm x 35 mm) is used, the diagonal sensor dimension is 43.27 mm. Therefore, mathematically speaking, the recommended lens focal length for this camera is 87 mm. But in practice, a 100 or 105 mm focal length lens is often used.

In order to achieve normal perspective, the lens focal length should remain constant from image to image. A compact camera with a zoom lens is not an ideal camera for oculoplastic photography because the lens focal length cannot be kept constant, unless the lens barrel has focal length parameters that can be used to shoot from the same focal distance using the rack and pull focus techniques.

There are two common types of digital cameras. They are interchangeable lens cameras and non-interchangeable lens cameras.

Interchangeable lens cameras:

1. Mirrorless digital camera

2. Digital single-lens reflex camera (DSLR)

Non-interchangeable lens cameras:

1. Compact camera

2. Bridge camera (single-lens reflex/SLR-like point-and-shoot camera)

3. Rangefinder-style camera

### Compact camera

A compact camera is also called a point-and-shoot camera. It is cheaper, lighter, and easy to use. It is often equipped with a zoom lens and a built-in flash. The image sensor is also smaller. This type of camera is not suitable for standardized photography because it is difficult to control the focal length from shot to shot.

### Mirrorless digital camera

A mirrorless digital camera has an interchangeable lens system as well as a large image sensor. This type of camera is usually slightly bigger than a typical compact camera but considerably smaller than a digital single-lens reflex camera. It has the ability to utilise a standard lens to capture undistorted images. This is a new camera system, and the range of lenses and external flash accessories is increasing.

### Digital single-lens reflex camera

Since digital single-lens reflex (DSLR) cameras have been around for longer, there are more accessories to choose from, making them the most versatile camera systems we have to date. This type of camera is the camera of choice for professional medical photographers.

### Which lens should I get?

The ideal lens to get is a dedicated macro lens [7]. A macro lens allows 1:1 reproduction of the object. A dedicated macro lens is always a prime lens with a fixed focal length. The focal length of a lens determines the angle of view of the camera. The focal length is often quoted in millimetres. A lens is regarded as wide angle when the focal length is less than 50 mm. A lens with a focal length greater than 50 mm is called a telephoto lens. Most compact cameras are fitted with zoom lenses, which are ideal for general-purpose photography. A zoom lens can alter its focal length, and it is made with more optical elements to achieve this. A prime lens on the other hand has a fixed focal length. This type of lens is made with fewer lens elements; therefore, it is capable of producing better quality photographs. When using a prime lens, the photographer has to physically move closer to the subject to increase magnification and vice versa. Since the lens focal length and sensor size combination are important for controlling perspective, the recommended lens for an APS-C sensor camera is a 60-mm macro lens. For a full-frame camera, the recommended lens is a minimum 90-mm macro lens. The working distance for these lens-sensor combinations is short enough to allow single-handed manipulation of eyelids.

### Lens calibration

The lens barrel of a dedicated macro lens is marked with linear scales. A 1:1 linear scale means the actual size of the subject is projected on the image sensor. Professional clinical photographers use linear scales like the Westminster scale of reproduction to maintain consistent magnification. This scale is based on the 35 mm film format. It is important to note that a macro lens mounted on an APS-C camera needs further calibration to achieve a field of view of a full-frame camera [10]. For example, the linear scale etched on the Canon EF-S 60 mm macro lens mounted on an APS-C sensor camera body does not match the Westminster scale on a full-frame (35 mm format) camera using a 100 mm macro lens. The calibrated lens will have an alternative linear scale, which corresponds to a full-frame camera. Young, et al. published the details of the calibration technique that involves photographing a ruler [11]. The horizontal dimension of a full-frame sensor is 36 mm. At 1:1 linear scale the photograph captured will show the image of the ruler at 36 mm. For an APS-C camera to achieve a field of view of a full-frame camera, the photographer has to physically move further away from the subject.

Here is a table (Table 1), containing the measurements of the image of the ruler [11] at various magnification ratios. The table compares three different cameras. At each magnification ratio setting, the amount of the ruler captured on the camera should correspond to these figures here. Due to the sensor size differences of the cameras listed here, the corresponding dimension of the image of the ruler will be different for a given magnification.

**Table 1:** A Comparison of the Measurements of the Ruler Image at Various Magnification Ratios Captured Using Three Cameras

Camera Models and Brand		Nikon D1x		Canon 5D Mark III		Canon 60D	
Sensor Size		23.7 mm		36 mm		22.3 mm	
Westminster Linear Scale	Size of Ruler Image Captured on Camera						
1:1	23.7		36		22.3		
1:1.5	35.6		54		33.45		
1:2	47.4		72		44.6		
1:3	71.1		108		66.9		
1:4	94.8		144		89.2		
1:6	142.2		216		133.8		
1:8	189.6		288		178.4		
1:12	284.4		432		267.6		
1:15	355.5		540		334.5		
1:20	474.0		720		446		
1:30	711.0		1080		669		
1:40	948.0		1440		892		
1:55	1303.5		1980		1226.5		
1:85	2014.5		3060		1895.5		

### Which lighting option should I go for?

A dedicated camera flash is the ideal set up. An electronic flash has a constant color temperature and the light intensity can be measured and adjusted manually to obtain a correct and consistent exposure.

Ambient light, which is often fluorescent with a mixture of natural daylight, should be avoided as their intensities and color temperature vary depending on the location of the patient in the room, time of day or weather. Most cameras have auto white balance correction built in, but the correction may not be consistent from shot to shot. An electronic flash has a consistent color temperature of 5500 K which is very near the natural daylight color temperature.

An electronic flash can be positioned in a consistent location to obtain a consistent image illumination [12]. A camera’s built-in flash can be useful in most situations except in 1:1 macro shots where the barrel of the lens may obscure the flash thus changing the direction and quality of the light.

A hot-shoe-mounted flashgun has a similar problem. Some professional medical photographers use custom-made brackets to mount the flashgun closer to the lens axis or simply hold the flash near the lens barrel. The flash has to be connected to the camera hot-shoe using a cable. The authors personally prefer a ring flash due to its portability and versatility. A ring flash is often used in the field of dentistry as it allows good illumination of oral cavities. A ring flash is a circular light source that is attached to the front of the lens [13]. This gives an even illumination on the subject to be photographed.

The benefit of using a ring flash is portability, and it allows single-handed operation of the camera. Modern ring flash output can be controlled to alter the ratios of light intensities on both halves of the ring. This increases contrast to demonstrate textured surfaces.

### Focusing grid

Most digital cameras are equipped with grid lines in the optical or electronic viewfinder. Grids help with composition by ensuring accurate alignment of the photographs [8]. It is possible to straighten an image in a computer but this will lead to loss of data from the original image.

### Colored background

It is important to set up a neutral-colored background to achieve a consistent look in the photographs [14]. The authors personally prefer a black background to avoid adding a color cast from colored backgrounds. However, it has its drawbacks as a black background makes it more difficult to achieve separation of dark-skinned subjects from the background using a single light source. We used a 1 m x 1 m piece of black polyester velvet mounted to the wall with reusable adhesive putty.

### Camera settings

Most interchangeable lens digital cameras allow the user to manually control the camera settings. Unlike the aperture priority and shutter priority modes, which are semi automatic modes, the manual mode allows the user to have complete control of the exposure setting. In this mode the photographer has to set the aperture, shutter speed, and ISO. Incorrect settings will result in blurred or incorrectly exposed images.

### Aperture

The aperture of a lens is the opening of the lens, which is analogous to the pupil of a human eye. The aperture is quantified by the F number, which is a ratio of the lens focal length and the diameter of the aperture. A wide aperture carries a small F-stop number. A small aperture is when the F-stop is 22 or more. In our practice, we use an aperture of F16 to F22 to achieve the desired depth of field and exposure. The depth of field is a 3D space, where any subject located within this space will be in focus. The depth of field is dependent on the aperture size [15], focal length of the lens, and the distance of the camera to the subject being photographed.

### Shutter speed

The shutter speed is a measure of how long the camera shutter blades are open to expose the sensor to the light. The slower the shutter speed, the more the light that is allowed to hit the image sensor [15]. A modern digital camera has a shutter speed range of 30 s to 1/8000th second. The shutter speed in flash photography mainly controls the brightness of the ambient light in the image. A slow shutter speed will allow more ambient light in the final image and vice versa. Since the ambient light in an ophthalmology outpatient clinic and theatre are not consistent in brightness and color temperature, we use a relatively high shutter speed, up to the maximum synchronisation speed (sync speed) to exclude ambient light in our clinical photographs. The sync speed differs in different cameras. A high camera sync speed allows the user to photograph at a higher shutter speed during flash photography. If the shutter speed is increased beyond the sync speed, the final image will have a black bar across the image. This is caused by the mechanical limitations of the shutter mechanisms. However, newer cameras overcome this barrier by introducing the high-speed sync mode where the flash output flickers rapidly to deliver an even exposure across the entire image.

### ISO

The light sensitivity of the image sensor is quantified by the ISO value. The ISO in a digital camera can be changed from shot to shot, unlike a film camera where the ISO can only be changed by changing the entire roll of film. The ISO of a digital camera can be altered by altering the level of amplification of the electrical signal generated by the complementary metal–oxide–semiconductor (CMOS) sensor. A high ISO is often used in dimly-lit situations where it is no longer possible to slow down the shutter speed or widen the aperture. A high ISO setting degrades image quality by introducing digital noise in the image. An image with a high level of digital noise typically has a grainy and pixelated appearance. Digital noise is also more prominent in small image sensors where the pixels are smaller.

### Electronic flash

The aperture, shutter speed, and sensor sensitivity (ISO) are the main parameters which determine the exposure of a given image [15]. However, in flash photography the exposure is also determined by the power of the flash output. The power of the flash output can be set manually or automatically by the camera. In the manual mode, one has to determine the base exposure with a test shot and adjust the flash power accordingly to achieve the correct exposure. The power output needs to be increased when the aperture is reduced or when the camera is moved further away from the subject. It is also possible for the camera to determine the flash power using the through-the-lens metering (TTL) function of the camera. In a TTL system, the camera fires a quick pre flash to determine the amount of light needed to illuminate the subject followed by the correct flash output to achieve the correct exposure. The TTL capability is reasonably accurate, therefore it is useful in a busy clinical situation where the speed of photography is important.

### File format

When an image sensor captures a photograph, the camera digital processor compiles the data into a file. Most digital cameras store images in JPEG format. A JPEG file is a compressed digital image file, which is favoured by non-professionals due to its small size [9, 16]. In order to achieve a small file size, some digital data is discarded during the file compression process. In some cameras, it is possible to store the photograph in a RAW file. A RAW file contains all the digital data from the image sensor. It is essentially the digital equivalent of a film negative. It is from this RAW file that the JPEG file is derived. A RAW file is significantly larger in size and needs to be converted to another file format like JPEG before it can be used for printing or display on the computer screen. We recommend the use of RAW file format as this allows the user to select the correct white balance to match the scene.

### White balance

White balancing is a process of removing unnatural color cast from an image. A camera’s auto white balance feature often has difficulty determining the color temperature of the scene leading to unrealistic blue or yellow color casts. When an image is captured in a JPEG format, the white balance setting, which is determined by the camera, is embedded in the compressed file format. There is very little room for further adjustment to correct any color cast at a later date. However, in a RAW file this white balance setting is not set and there is still room for color correction in a computer. In situations where accurate color reproduction is critical, it is possible to standardise the white balance by creating a customised color profile for the camera. There are specialised color charts like the X-rite ColorChecker Passport (X-Rite Inc., Grand Rapids, MI) to create custom color profiles [17].

### The optimal camera setting for oculoplastic photography

To obtain an adequate exposure with the exclusion of ambient lighting, a sufficient depth of field to get everything in focus, and the least amount of digital noise, the following camera settings indicated in Table 2 are used.

**Table 2:** Optimal Camera Setting for Oculoplastic Photography

Aperture	f16-f22
Shutter speed	1/250 s (or up to the max sync speed)
ISO	100-200
File format	RAW
Flash Output	Automatically set by camera using eTTL
White balance	Flash mode or 5500 K (accurate and customised white balance can be applied in post processing in a computer)

### How to capture images

**Figure 2:**
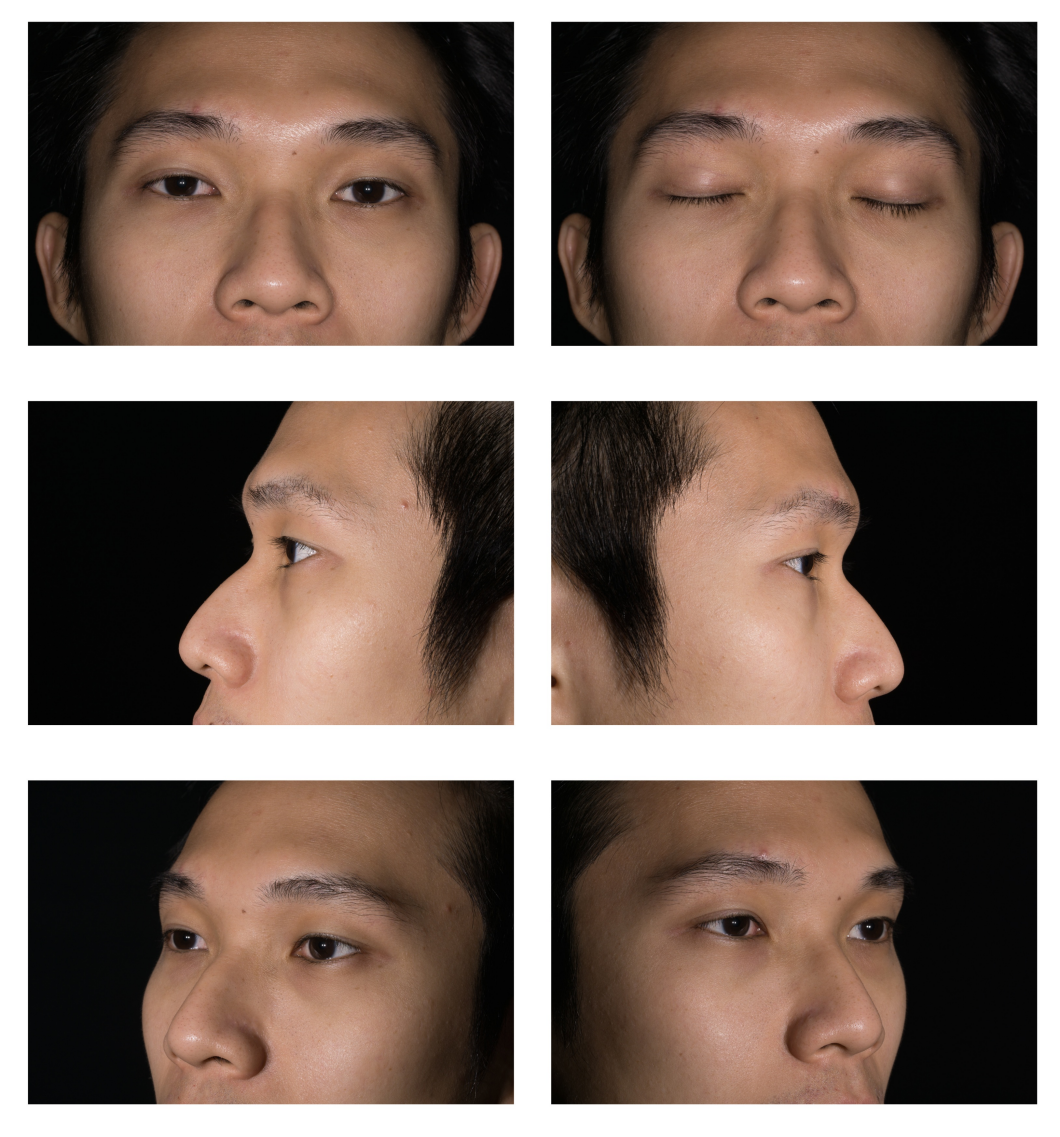
Standard Eyelid at 1:4

**Figure 3:**
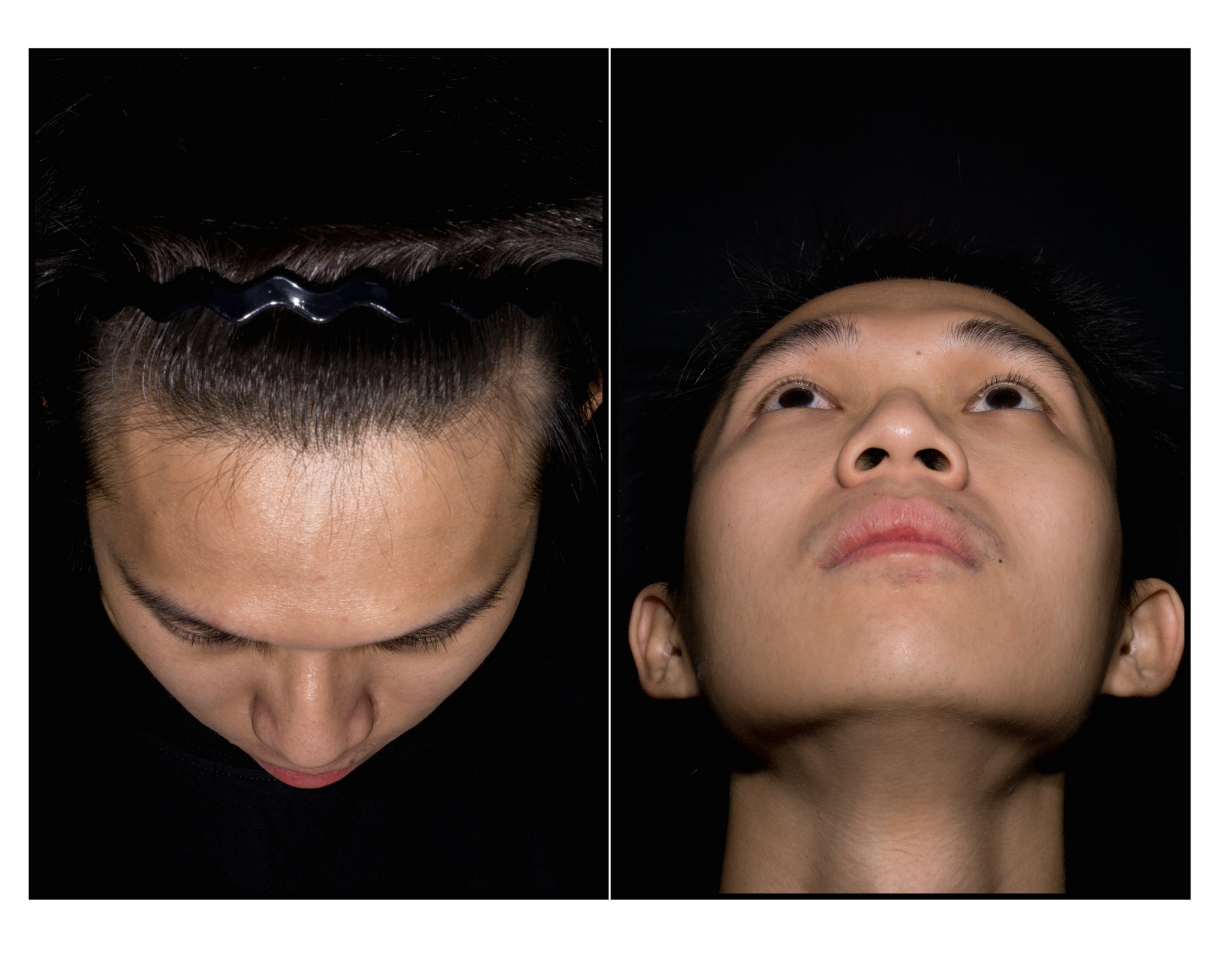
Superior and Inferior at 1:4

**Figure 4:**
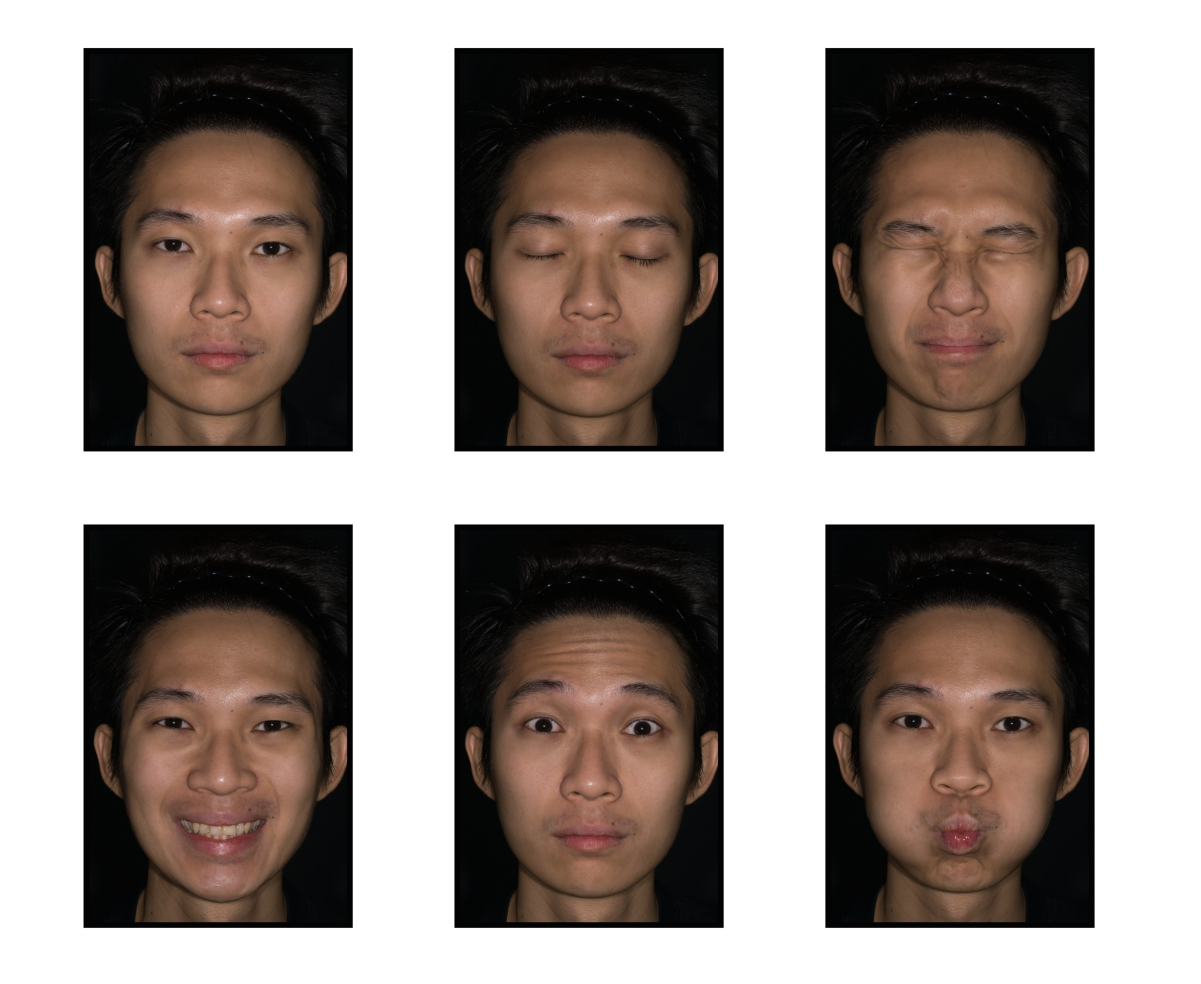
Full Face Views at 1:8

Informed written consent should be obtained prior to clinical photography. A photograph of the consent form should be taken before taking patient photographs. This step ensures that the images that follow are correctly tagged. Informed written consent was obtained from the model who posed for the photographs in this study. Informed written consent was also obtained for publication of the photos in a journal. The camera is set to manual focus because the auto focus mechanism will reset the pre-calibrated lens [18]. In order to focus, one has to physically move the camera forward and backward until the image on the viewfinder is sharp. The following standardised poses are recommended at various magnification ratios, as illustrated in Figures 2-4. The photographs taken using a calibrated lens at these magnification ratios are consistent without requiring further editing or cropping, as shown in Table 3.

**Table 3:** Recommended Standardized Poses at Various Magnification Ratios

Views and Magnification Scales	Examples of Clinical Conditions
Standard Eyelid 1:4	Eyelid tumors, eyelid malpositions, and following surgery
Superior and Inferior 1:4	Proptosis, Enophthalmos
Facial 1:8	Facial palsy, facial or cheek lesions

### Digital workflow: how to process the image files from the camera

Digital asset management is an important part of clinical photography. This process ensures that the images are systematically renamed, stored, and encrypted for ease of retrieval and for data protection.

### Folder and file naming convention

Once an image is captured in a camera, the files should be downloaded straightaway into a computer and backed up straightaway in another hard drive [16, 19]. There are various computer software programs that facilitate the process of image download, storage, and retrieval. We organise our images in chronological order in separate folders. The folders are named by the date in a reverse numerical format starting with the year followed by the month and date. This is to ensure that the computer displays the folders in a chronological order instead of the default alphabetical order. For example 1 January 2014 is written as 20140101. This folder naming convention also applies to the file names to ensure the file names are not duplicated. Most RAW file processing software programs automate the input and file renaming process. To ensure data security and patient anonymity, try to avoid using patient names within the file.

### Image file tagging with keywords

The images are then tagged in batches with keywords including a brief clinical history to allow the search engine to retrieve the images at a later date. This information is stored in the image metadata. Copyright details can also be included within the metadata. The metadata is sometimes embedded within the image file and is searchable on the computer or the internet. The software programs that offer these functionalities are Adobe Lightroom and Apple Aperture. These programs are used by professional photographers, and they are capable of handling large numbers of raw image files.

### Data security

In order to comply with the data protection act, the image should be kept in a secure device and location, kept up-to-date, and backed up regularly. We recommend keeping three identical copies of the data in hard disks. The third copy of the data should be kept in a separate and secure location. The data across three hard disks can be synchronised with commercial back up or a disk cloning software. It is also important to protect the data within the memory cards by regularly downloading it into secure hard disks and formatting it regularly [20]. Since there is no data encryption function within any camera available to us today, memory cards used for patient photography should never be taken outside the clinical setting or the secure premises. There are many software programs that can recover deleted data or overwritten data; therefore, any unwanted hard disks and memory cards previously used for storing patient data should be physically destroyed to dispose the information permanently.

## Conclusions

The choice of photographic equipment for use in oculoplastic photography is very specific. A broad understanding of the types of camera, flash, and lenses will help one choose the correct system for this purpose [21].
